# What Is Going on with Childhood?

**DOI:** 10.3390/bs13080671

**Published:** 2023-08-10

**Authors:** Luca Cerniglia

**Affiliations:** Faculty of Psychology, International Telematic University Uninettuno, 00185 Rome, Italy; luca.cerniglia@uninettunouniversity.net; Tel.: +39-066920761

Several studies have shown that the age of onset of psychopathology is decreasing, and that at least some clinical manifestations (e.g., eating disorders) that once typically belonged to adolescence may present themselves much earlier [1,2]. The articles composing this Special Issue will help clarify the mechanisms underpinning this phenomenon; however, generally speaking, this fact may be related to sociological, psychological, and even biological aspects, or more convincingly to the interaction of these issues. The duration of developmental stages itself is being questioned, with a shortening of childhood and an extension of adolescence as well as young adulthood.

From a sociological standpoint, scholars are reconsidering the concept of childhood, modifying its historical perception of an enchanted time, free from care and responsibilities. Several labels, such as hurried and toxic childhood, have emerged, indicating a growing consensus that this developmental phase is undergoing significant changes [3]. Some studies have focused on the links between children’s exposure to adult imagery through social media and their subsequent imitation of adult behaviors. This phenomenon is referred to as the disappearing child, where the boundaries between childhood, adolescence, and adulthood are blurred, and children are asked to adopt adult-like behaviors [4]. Building on the disappearing childhood argument, other scholars have introduced the concept of the hurried child, examining how children are pressured into assuming adult perspectives in various contexts [5]. This rush into adulthood has been associated with negative consequences, including experimentation with drugs and alcohol, precocious sexual behavior, psychopathology, and suicide.

From a psychological point of view, the crisis of contemporary childhood has also been linked to parenting practices, particularly the concept of hyper-parenting [6]. Hyper-parenting refers to the overscheduling of children in after-school activities as parents strive to provide their children with a competitive edge. This hyper-scheduling places increased pressure on children, compromising their health and perceived happiness. In parallel to the discussion of childhood crises, the concept of adultification has been introduced. Adultification refers to situations where children’s thoughts, roles, or activities resemble those of adults [7]. This phenomenon can encompass ‘precocious knowledge’ or children being prematurely exposed to adult life perspectives. Adultification is also observed when children assume adult roles within the family, taking on caregiving responsibilities beyond the norms of their age. Research also indicates that early life experiences, particularly the parent–child relationship, can significantly impact lifelong mental health. Dysfunctional caregiving, such as neglect, parental mental illness, and familial violence, has been strongly associated with mental illness in children and adults [7].

From a biological perspective, it has been observed that the early onset of psychopathological symptoms is frequently associated with precocious pubertal development. Specifically, early puberty in girls has been associated with a higher likelihood of disordered eating one year later [8,9,10,11]. Moreover, both early-maturing girls and boys have a higher incidence of bulimic behavior compared to their on-time or late-maturing peers [12]. Life history theory, the psychosocial acceleration hypothesis, child development theory, and the adaptive calibration model posit that early experiences could program the length of childhood and the age of pubertal transition [7]. Specifically, these theories suggest that slower developmental strategies would be advantageous in conditions of low stress, allowing an individual to absorb information from social and family environments early in life that may increase survival and parenting skills before transitioning into the reproductive stage of development. In contrast, faster developmental strategies, allowing for earlier reproduction, are proposed to increase reproductive success and fitness in contexts of adversity where long-term survival is uncertain. This is posited to be the case of the uncertain and unreliable environment that children nowadays experience [13].

It is undeniable that a transformation is occurring in youths, both on a sociopsychological level and also in some biological aspects, with children and/or preadolescents showing emotional/behavioral functioning typical of later stages and presenting early physical maturation. On the other hand, clinical experience is suggesting that a growing number of girls do not have menarche until late adolescence (if ever). These anecdotal pieces of evidences seem associated with a sort of emotional and biological dysregulation [14,15,16,17,18,19] that should be thoroughly and systematically studied to unveil the mechanisms that govern it.
